# Effects of Lactate Transport Inhibition by AZD3965 in Muscle-Invasive Urothelial Bladder Cancer

**DOI:** 10.3390/pharmaceutics15122688

**Published:** 2023-11-28

**Authors:** Ana Silva, Ana Félix, Mónica Cerqueira, Céline S. Gonçalves, Belém Sampaio-Marques, Adhemar Longatto-Filho, Fátima Baltazar, Julieta Afonso

**Affiliations:** 1 Life and Health Sciences Research Institute (ICVS), University of Minho, Campus of Gualtar, 4710-057 Braga, Portugal; id10583@alunos.uminho.pt (A.S.); mafaldafelix98@gmail.com (A.F.); id10586@alunos.uminho.pt (M.C.); celinegoncalves@med.uminho.pt (C.S.G.); mbmarques@med.uminho.pt (B.S.-M.); longatto@med.uminho.pt (A.L.-F.); fbaltazar@med.uminho.pt (F.B.); 2 ICVS/3B’s—PT Government Associate Laboratory, 4710-057 Braga, Portugal; 3 Laboratory of Medical Investigation (LIM14), Faculty of Medicine, São Paulo State University, São Paulo 01049-010, SP, Brazil; 4 Molecular Oncology Research Center, Barretos Cancer Hospital, Barretos 14784-400, SP, Brazil

**Keywords:** urothelial bladder cancer, Warburg effect, lactate, monocarboxylate transporters, AZD3965

## Abstract

The Warburg Effect is characterized by high rates of glucose uptake and lactate production. Monocarboxylate transporters (MCTs) are crucial to avoid cellular acidosis by internal lactate accumulation, being largely overexpressed by cancer cells and associated with cancer aggressiveness. The MCT1-specific inhibitor AZD3965 has shown encouraging results in different cancer models. However, it has not been tested in urothelial bladder cancer (UBC), a neoplasm where rates of recurrence, progression and platinum-based resistance are generally elevated. We used two muscle-invasive UBC cell lines to study AZD3965 activity regarding lactate production, UBC cells’ viability and proliferation, cell cycle profile, and migration and invasion properties. An “in vivo” assay with the chick chorioallantoic membrane model was additionally performed, as well as the combination of the compound with cisplatin. AZD3965 demonstrated anticancer activity upon low levels of MCT4, while a general lack of sensitivity was observed under MCT4 high expression. Cell viability, proliferation and migration were reduced, cell cycle was arrested, and tumor growth “in vivo” was inhibited. The compound sensitized these MCT4-low-expressing cells to cisplatin. Thus, AZD3965 seems to display anticancer properties in UBC under a low MCT4-expression setting, but additional studies are necessary to confirm AZD3965 activity in this cancer model.

## 1. Introduction

Cancer remains the second deadliest disease worldwide, and bladder malignancies stand as the 10th most common to be diagnosed, accounting for 573,000 new cases and 213,000 associated deaths in 2020 [1]. Roughly 90% of all bladder tumors arise from urothelial cells, and these are more prevalent in developed countries and four times more common in men. Tobacco and occupational exposure to carcinogenic agents are the most relevant risk factors, making elderly people more susceptible due to long-term exposure [2,3]. Representing 75% of all cases, non-muscle-invasive bladder cancer is the most frequently diagnosed subtype of urothelial bladder carcinoma (UBC), being usually treated with transurethral resection; once recurrence rates are high, this treatment is generally complemented with intravesical chemotherapy or immunotherapy [4,5]. The remaining 25% of the tumors are originally muscle-invasive (MI-UBC), standardly undergoing radical cystectomy and pelvic lymphadenectomy. Due to early systemic dissemination, cisplatin-based chemotherapy is largely recommended, but the patients suffer from high rates of therapy resistance, morbidity and mortality [6,7,8]. The five-year survival rate ranges from 98% in early stages to 7% upon disseminated disease [9]. Due to the recurrent/progressive nature of this disease and the development of primary/acquired cisplatin-resistance, UBC patients represent a tremendous burden to healthcare systems [10]. New therapeutic approaches, namely, immune checkpoint inhibitors [11] and Erdafitinib targeted therapy [12], have been approved, but cisplatin-based chemotherapy stands as first line option for MI-UBC-eligible patients [13]. While multiple authors have attempted to decipher the molecular mechanisms underlying UBC chemoresistance [14,15,16], novel chemical templates that can concurrently tackle drug resistance and treat the disease are urgently needed to improve treatment outcome and survival expectancy, as well as reduce morbidity.

High levels of glucose consumption, ultimately degraded to lactate in an accelerated glycolytic process independent of oxygen availability, help to support cancer cells’ energy demands for their rapid growth rates in what is known as the Warburg effect [17,18]. The shift from oxidative phosphorylation (preferred by the majority of healthy cells) to glycolysis leads to the production of copious amounts of lactate [19]. In UBC, as in many other malignancies, this process relies on the upregulation of key players in the glycolytic pathway, as glucose transporters (GLUTs), hexokinase isoform 2 (HK2) and lactate dehydrogenase isoform A (LDHA), which are nowadays viewed as targets for novel therapeutic approaches [20]. Additionally, and to avoid cell death due to the accumulation of lactate, cancer cells also upregulate monocarboxylate transporters (MCTs) to extrude the molecule. Among all isoforms, MCTs 1 and 4, together with their chaperone CD147, are largely overexpressed by cancer cells and associate with cancer aggressiveness [21].

MCT1, a bi-directional proton-mediated shuttle protein, can serve as a possible biomarker of poor prognosis in different types of cancers [22]. MCT1 is overexpressed in bladder cancer and has been strongly associated with the maintenance of the glycolytic phenotype of tumors and with cisplatin resistance [14,15,23]. Once it is considered one of the most important regulators of lactate shuttling, and in order to disrupt this process and compromise the proliferative effectiveness and survival of cancer cells, the pharmacological inhibition of MCT1 has been attempted. AZD3965 is a specific MCT1 inhibitor developed by Astrazeneca, currently in phase I/II clinical trial for solid tumors and lymphomas [24,25]. Preclinical “in vitro” studies concluded that AZD3965 was able to decrease lactate shuttling and proliferation rates in cell lines with low MCT4 expression levels [26,27]. “In vivo” treatment through oral gavage decreased tumor growth in both mono and combined therapy (with chemotherapeutic agents or radiotherapy) in lymphoma, lung, breast, kidney, head and neck, and colon cancers; combined therapy was even able to promote tumor regression [28]. Its safety has been preclinically demonstrated [29], and a phase I dose-escalation study (included on the ongoing clinical trial) revealed only grade 1 and/or 2 treatment-emergent adverse events (retinopathy, fatigue, anorexia, and constipation) [25].

To the best of our knowledge, AZD3965 has not yet been tested in bladder cancer settings. Thus, the aim of this study was to perform a preclinical evaluation of AZD3965 activity in advanced UBC, both in monotherapy and in combination with cisplatin, using MI-UBC cell lines in “in vitro” and “in vivo” assets. The anticancer effect of AZD3965, as well as sensitization to cisplatin, was demonstrated in a MI-UBC cell line with low levels of MCT4, while under a high MCT4 setting the opposite results were observed.

## 2. Materials and Methods

### 2.1. Cell lines and Conditions for Cell Culture 

Four muscle-invasive bladder cancer cell lines—HT1376R, 253J, BFTC-905 and TCCSUP—were used in this study. HT1376R is a cisplatin-resistant HT1376 subline (termed HT1376rCDDP1000) obtained from the Resistant Cancer Cell Line (RCCL) collection (www.wass-michaelislab.org/rccl.php, accessed on 2 May 2017) [30]; cisplatin resistance was accomplished by continuously exposing the parental HT1376 cell line to increasing concentrations of cisplatin, as previously described [31]. For maintenance of the selection pressure, HT1376R cells were grown in the presence of cisplatin 1 µg/mL. HT1376, 253J, BFTC-905 and TCCSUP cells were acquired from ATCC (American Type Culture Collection) and properly authenticated by short-tandem repeat assessment. Cell culture was performed in IMDM (Iscove’s Modified Dulbecco’s Medium, PAN-BioTech^TM^, Aidenbach, Germany) medium supplemented with 1% antibiotics (penicillin/streptomycin solution, GRiSP, Porto, Portugal) and 10% heat-inactivated fetal bovine serum (FBS, PAN-BioTech^TM^, Aidenbach, Germany) (unless otherwise specified), in a humidified incubator (37 °C, 5% CO_2_). Cells were counted in a Neubauer chamber for density calculation. 

AZD3965 was obtained from Cayman Chemical (Ann Arbor, MI, USA); stock solutions of 100 mM in 0.01% DMSO were prepared and stored at −20 °C. Working solutions of cisplatin ((CDDP, cis-diamminedichloroplatinum(II)) were prepared from stock solutions of 1 mg/mL in 10% NaCl, kindly provided by the Pharmaceutical Services of the Portuguese Institute of Oncology, Oporto, Portugal. To avoid cisplatin acute effects that could induce bias in the results, the drug was removed from the culture medium three passages before performing the experiments.

### 2.2. Protein Extraction and Western Blot

To analyze basal protein expression, all cell lines were grown in 6-well plates, in complete culture medium, until 90–100% confluence. To analyze the effect of AZD3965 in HT1376R and 253J cells, these cell lines were grown overnight in 6-well plates and treated with the respective compound concentration (10 nM, 100 nM and 1000 nM) or control condition (0.0 nM AZD3965 in drug vehicle, 0.01% DMSO) for 24 or 48 h. To extract protein, a first wash with PBS 1× was performed, followed by treatment with lysis buffer (supplemented with protease inhibitor cocktail, Roche Applied Sciences, Penzberg, Germany). Cells were then collected by scrapping and incubated for 10 min on ice. Afterwards, suspensions were centrifuged at 13,000 rpm for 15 min at 4 °C. The supernatant was collected, and protein content was quantified by the Bradford assay (Sigma-Aldrich^®^, St. Louis, MO, USA). In total, 20 µg of total protein of each sample was separated in a 10% polyacrylamide gel by SDS-PAGE at 100 V for 90 min and transferred (Trans-Blot^®^ Turbo^TM^ Transfer System, Bio-Rad Laboratories, Hercules, CA, USA) to a nitrocellulose membrane (25 V for 30 min). Membranes were blocked in 5% milk with 1× TBS-T for 1 h prior to overnight incubation with specific primary antibodies (and loading controls) at 4 °C (Table S1). Membranes were then washed with TBS-T and incubated with specific secondary antibodies at room temperature (RT) under agitation (Table S1). The washing procedure was repeated, and chemiluminescence (WesternBright^®^ Sirius^®^, Advansta, San Jose, CA, USA) was used to detect immunoreactive bands on a Sapphire^TM^ Biomolecular Imager (Azure Biosystems, Dublin, OH, USA).

### 2.3. Immunofluorescence

All cell lines were seeded (5 × 10^4^/well) in complete IMDM medium, in 12-well plates containing coverslips. Cells were fixed and permeabilizated with cold methanol at −20 °C for 20 min and blocked for 30 min at RT in 5% BSA. Cells were incubated overnight (at 4 °C) with MCT1, MCT4 and CD147 primary antibodies (Table S1). After washing, cells were incubated with fluorescence-conjugated secondary antibodies (Table S1) for 1 h, at RT, in the dark. Cells were mounted with Fluoroshield^TM^ plus DAPI (4’,6-diamidino-2-phenylindole, Sigma-Aldrich^®^, St. Louis, MO, USA), and images were obtained by fluorescence microscopy (Olympus^®^ BX61 fluorescence microscope, Tokyo, Japan).

### 2.4. Quantification of Extracellular Lactate Levels by Colorimetry

Extracellular levels of lactate at 24 and 48 h post AZD3965 treatment were quantified to assess cell metabolism. Cells were plated in triplicate in 48-well plates (7.5 × 10^4^ cells/well) and allowed to adhere overnight in complete IMDM. After removing the medium, cells were treated with AZD3965 (0.1 nM–10,000 nM) or control condition in incomplete (without FBS) IMDM. Extracellular lactate was quantified from collected medium at the end of each time-point using a commercial colorimetric kit (Spinreact, Girona, Spain), following the manufacturer’s instructions. Biomass was assessed by the Sulforhodamine B (SRB) assay (TOX-6, Sigma-Aldrich^®^, St. Louis, MO, USA), and readings were performed at 490 nm (Varioskan^®^ Flash spectrophotometer, Thermo Fisher Scientific, Waltham, MA, USA). The results (from at least three independent assays, each in triplicate) were expressed in total µg/biomass (GraphPad 8.4.2. software). 

### 2.5. High-Performance Liquid Chromatography (HPLC)

Cell metabolism was also assessed in HT1376R and 253J by quantification of the extracellular levels of lactate and glucose at 24 and 48 h post AZD3965 treatment by HPLC. Cells were plated in triplicate in 24-well plates (1 × 10^5^/well) and allowed to adhere overnight in complete IMDM. Medium was then removed, and cells were treated with AZD3965 (10 nM, 100 nM and 10,000 nM) or control condition in incomplete IMDM. At the end of each time-point, spent medium was collected and stored at −20 °C. Lactate and glucose concentrations in the samples were then determined by HPLC, using a refractive index detector IOTA2 (Gilson, Middleton, WI, USA) and a carbohydrate H^+^ column (SS-100, H^+^, 8 µm, Thermo Scientific™ Hypersil, Thermo Fisher Scientific, Waltham, MA, USA), at 54 °C. A solution of H_2_SO_4_ (0.0025 M) was used as the mobile phase at a flow rate of 0.7 mL/min. The results from at least three independent assays, in triplicate, were expressed in mM, using the GraphPad 8.4.2. software.

### 2.6. Assesment of Mithocondrial Activity

Mitochondrial activity upon AZD3965 treatment was assessed in HT1376R and 253J at 24 and 48 h of exposure to the compound by Mitotracker labelling. Cells were plated in 24-well plates (2 × 10^5^/well) and allowed to adhere overnight in complete IMDM. Medium was then removed, and cells were treated with AZD3965 (10 nM, 100 nM and 10,000 nM) or control condition. At the end of each timepoint, cells were incubated with 200 nM of Mitotracker Red (membrane polarization; M7512, Invitrogen^TM^, Waltham, MA, USA) and Mitotracker Green (mitochondrial mass; M7514, Invitrogen^TM^, Waltham, MA, USA) probes for 2 h. After incubation, cells were collected, washed with PBS 1×, centrifuged (1500 rpm, RT) and resuspended in FACs Buffer 1x. Fluorescence signal acquisition was performed using FACS LSRII flow cytometer (BD Biosciences^®^, San Jose, CA, USA), considering at least 20,000 events. The FlowJo software (version 10, Tree Star, Inc, San Francisco, CA, USA) was used to extract the results from at least three independent assays. Mitochondrial activity was determined through Mitotracker Red/Mitotracker Green (polarization/mass) ratio in cells positively stained with both probes. Results were then analyzed with GraphPad 8.4.2. software.

### 2.7. Bioluminescence ATP Quantification Assay

To analyze the effect of AZD3965 in ATP production by HT1376R and 253J cells, these cell lines were grown overnight in 6-well plates (1 × 10^6^ cells/well) and treated with the respective compound concentration (10 nM, 100 nM and 1000 nM) or control condition for 48 h. Cell lysates were then obtained, as previously described. ATP content was analyzed in the cell lysates, using a bioluminescence assay for the quantitative determination of ATP with recombinant firefly luciferase and its substrate D-luciferin, according to the manufacturer’s instructions (Molecular Probes^TM^, Eugene, OR, USA). Luminescence was measured in a hybrid multi-mode microplate reader with luminescence detection mode (Synergy^TM^ H1, Biotek, Winooski, VT, USA). Results were normalized to the protein content, assessed by the Bradford assay (Sigma-Aldrich^®^, St. Louis, MO, USA), as previously described.

### 2.8. Cell Viability Assay

Cell viability after AZD3965 treatment was evaluated on both mono-(AZD3965 only) and combined therapy (AZD3965 + cisplatin). After seeding of HT1376R and 253J cells in triplicate (7.5 × 10^4^/well) in 48-well plates and overnight adhesion, cells were exposed to different compound concentrations for 24, 48 and 72 h (monotherapy) or 72 h (combined therapy). Compound solutions were prepared in incomplete IMDM culture medium. Regarding AZD3965 monotherapy, cells were treated with a range of AZD3965 concentrations (0.1 nM–10,000 nM). In the combined treatment, cells were treated with previously determined IC_50_ doses of cisplatin in 10% NaCl (4.71 µg/mL for HT1376R cells [32] and 1.59 µg/mL for 253J cells (unpublished results)), which were combined with 10, 100 and 1000 nM AZD3965; controls were performed with 0.01% DMSO + 10% NaCl in incomplete medium. The cytotoxic effect was evaluated by the SRB assay (TOX-6, Sigma-Aldrich^®^, St. Louis, MO, USA). Absorbance was read at 490 nm (Varioskan^®^ Flash, Thermo Fisher Scientific, Waltham, MA, USA). Results were expressed as the mean percentage ± SD of viable cells normalized to the control (considered as 100% viability). Graphical analysis of at least three independent experiments (each one in triplicate) was performed through GraphPad Prism 8.4.2 software.

### 2.9. Cell Proliferation Assay

HT1376R and 253J cells were seeded in 96-well plates (2 × 10^5^ cells/well (24 h), 1.5 × 10^5^ cells/well (48 h) and 1 × 10^5^ cells/well (72 h) for both cell lines) and incubated overnight in complete IMDM for cell adhesion. Compound concentrations and treatment conditions were similar to those used in the cell viability assay (Section 2.5). Cell proliferation was quantified using a commercial colorimetric kit (Cell Proliferation ELISA, BrdU, Roche Applied Sciences, Penzberg, Germany). Following the incubation period, cells were stained with 20 µM of BrdU labeling solution and reincubated for 8 h. Cells were then fixed, followed by DNA denaturation and incubation with anti-BrdU-POD antibody (1:100 dilution) for 90 min, at RT. Cell staining was achieved with TBM (tetramethyl-benzidine) for 5–30 min; the reaction was then stopped with 1 M H_2_SO_4_. Absorbances were measured at 450 nm (Varioskan^®^ Flash, Thermo Fisher Scientific, Waltham, MA, USA), and the percentage of cell proliferation was normalized to the control condition. Graphical analysis of at least three independent experiments (each one in triplicate) was performed through the GraphPad Prism 8.4.2 software.

### 2.10. Wound-Healing Assay

HT1376R and 253J cells were seeded in 6-well plates and incubated until 90% confluence was reached. Two scratches were performed in each well using plastic micropipette tips for wound opening. After gentle washing with PBS 1×, cells were treated with AZD3965 (10, 100 and 1000 nM) or control condition in incomplete medium. At 0, 24 and 48 h, specific wound sites (4 reference points for each wound) were photographed at 100× magnification (Olympus^®^ IX51, Tokyo, Japan). Migration distance was calculated with the beWound–Cell Migration Tool (Version 1.5) software. Percentage of cell migration (normalized to the control condition) was assessed by the GraphPad Prism 8.4.2. software.

### 2.11. Cell Invasion Assay

Invasion capacity of HT1376R and 253J cells was evaluated using a 12-well Transwell invasion assay in BioCoat™ Matrigel^®^ Invasion Chambers (Corning^®^, New York, NY, USA). Complete IMDM (chemoattractant) was added to the bottom of each well. Inserts containing 5 × 10^4^ cells in incomplete IMDM with control condition or 100 and 1000 nM AZD3965 were placed in the wells. After 72 h, non-invading cells were removed, and invading cells were washed in PBS 1×, fixed with cold methanol and stained with Giemsa. Membranes were photographed under a stereomicroscope (Olympus^®^ S2 × 16, Tokyo, Japan). The ImageJ software version 1.50i was used to count the invading cells, and the percentage of invading cells (normalized to the control condition) was assessed by the GraphPad Prism 8.4.2. software.

### 2.12. Cell Cycle Analysis

Cell cycle analysis was performed on HT1376R and 253J cells stained with propidium iodide (PI) by flow cytometry. The cell lines were cultured in completed IMDM, in T25 flasks (3.75 × 10^4^ for 253J and 2 × 10^4^ for HT1376R), and allowed to adhere overnight. Cells were then treated with 100 nM or 1000 nM of AZD3965, or control condition, in complete medium for 24, 48 and 72 h. After collecting, pelleting and washing floating and adherent cells with PBS 1×, they were fixed with cold 70% *v*/*v* ethanol (30 min, 4 °C). Then, samples were stained with PI solution (20 μg/mL of PI (Invitrogen^TM^, Waltham, MA, USA) + 250 μg/mL of RNAse (Invitrogen^TM^, Waltham, MA, USA) diluted in 0.1% Triton X-100 in PBS 1×) and incubated for 1 h in the dark at 50 °C, under agitation (400 rpm). Stained cells were analyzed by flow cytometry (FACS LSRII cytometer, BD Biosciences^®^, San Jose, CA, USA), considering at least 20,000 events. Cell cycle distribution (from at least three independent assays) was analyzed by the FlowJo software (version 10, Tree Star, Inc., San Francisco, CA, USA).

### 2.13. Chick Chorioallantoic Membrane (CAM) Assay

Fertilized chicken eggs (supplied by Pinto Bar, Amares, Braga, Portugal) were incubated, horizontally, in an 80% humidified incubator, at 37 °C. On day 3 post-incubation, a small window was opened in the eggshell; eggshell was then sealed, and the eggs were reincubated. On day 9, 2 × 10^6^ of HT1376R cells was mixed with 10 μL Matrigel (Corning^®^, New York, NY, USA) and grafted on the CAM; windows were closed, and the eggs were reincubated. On day 13, tumors were photographed “in ovo” and treated with 100 nM or 1000 nM AZD3965 (or control condition), diluted in incomplete IMDM (total volume: 10 μL); windows were reclosed, and the eggs were incubated again. On day 17, tumors were photographed “in ovo”. After embryo anesthesia (CO_2_ for 20 min) and sacrifice (−80 °C for 20 min), the tumor-containing CAMs were collected from each egg, fixed in 4% formaldehyde and photographed “ex ovo” under a stereomicroscope (Olympus^®^ S2 × 16, Tokyo, Japan). ImageJ (version 1.50i) was used to obtain tumor perimeter and total number of blood vessels, for quantification of tumor growth and angiogenesis occurrence. Results were analyzed with the GraphPad Prism 8.4.2 software.

### 2.14. Immunohistochemistry

Representative 4 μm thick tissue sections from excised tumors derived from the CAM assay were used to conduct immunohistochemistry, which allows for the semi-quantitative evaluation of proliferation and angiogenesis occurrence in the tumors using specific antibodies (anti-Ki67 and anti-lectin, respectively). Immunoreactivity was detected with the Thermo Scientific™ Lab Vision™ UltraVision™ detection kits, ONE Detection System: HRP Polymer (Ki67) and Large Volume Detection System: antiPolyvalent, HRP (lectin) (Thermo Fisher Scientific, Waltham, MA, USA). Primary antibodies were used (Table S1); negative controls were performed by omitting primary antibody staining. A pathologist semi-quantitatively evaluated protein expression, according to the following grading system: 0 for 0%, 1 for 0–10%, 2 for 10–50% and 3 for over 50% of positive cells. 

### 2.15. Statistical Analysis

Results from “in vitro” and “in vivo” assays were analyzed by Student’s *t*-test or Two-Way Anova, using the GraphPad Prism 8.4.2 software. Results are presented as normalized means ± standard error of the mean (SEM) (unless otherwise specified), from at least three independent experiments. A *p* value < 0.05 was considered significant (* *p* < 0.05, ** *p* < 0.01, *** *p* < 0.005 and **** *p* < 0.001).

## 3. Results

### 3.1. Impact of MCT1 Inhibition by AZD3965 in Muscle-Invasive UBC Cells

**Figure 1:**
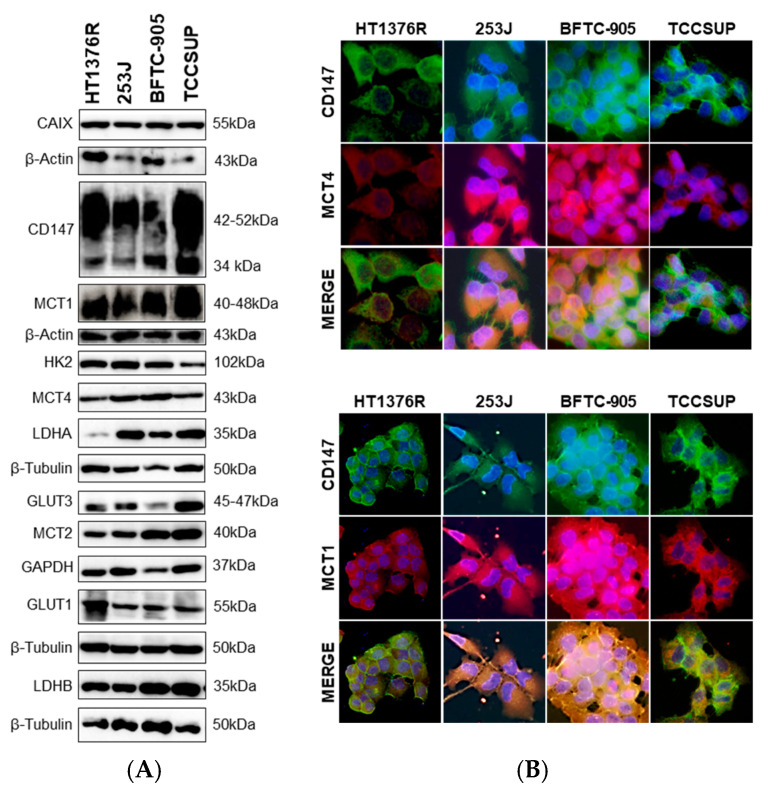
Western blot (**A**) and immunofluorescence (**B**) results showing expression of glycometabolism-related biomarkers in muscle-invasive urothelial bladder carcinoma cell lines (HT1376R, 253J, BFTC-905 and TCCSUP). Western blot (**A**) represents similar blots from three independent cell lysates (shown in Figure S1; quantification of the results shown in Figure S2); β-Actin, β-Tubulin and GAPDH (glyceraldehyde 3-phosphate dehydrogenase) were used as loading controls. Immunofluorescence images (**B**) show co-localization of selected biomarkers; pictures were captured at 200× amplification.

In order to evaluate the impact of AZD3965 treatment in UBC cell lines, we firstly assessed the expression of MCT isoforms, their chaperone CD147 and other glycometabolism-related proteins in four MI-UBC cell lines by Western blot (Figure 1A) and immunofluorescence (Figure 1B). The biomarkers were expressed by the cell lines at various levels (quantification of the results in Figure S2). TCCSUP cells expressed higher levels of MCT1 than the remaining cells, while HT1376R expressed lower levels of MCT4; immunofluorescence confirmed MCT1 and MCT4 co-localization with CD147, on the one hand, and the lower staining intensity of MCT4 in HT1376R cells on the other hand. High levels of GLUT1 and MCT2 were seen in HT1376R and 253J cells, respectively. Given the apparent inverse pattern of expression between these two cell lines, we decided to further perform the functional assays with these cells.

**Figure 2:**
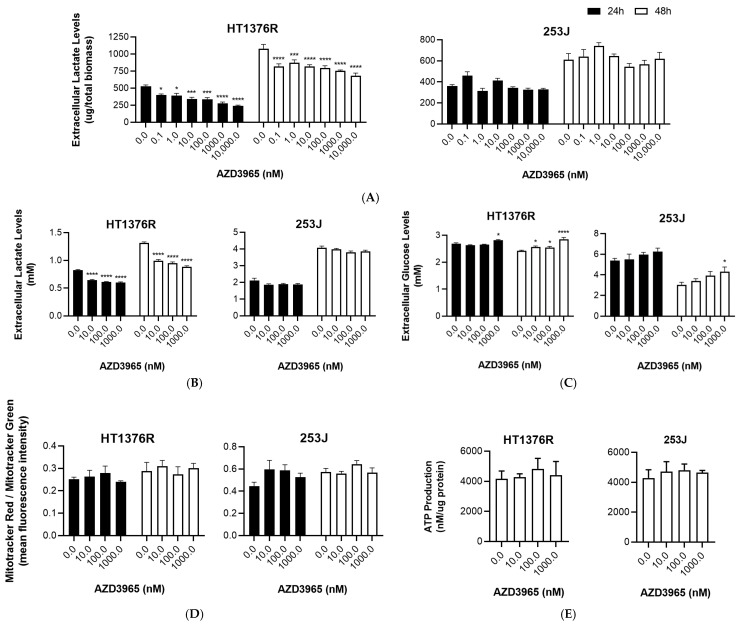
Effect of AZD3965 on metabolic functions of HT1376R and 253J urothelial bladder carcinoma cell lines, detected by quantification of lactate (**A**,**B**) and glucose (**C**) levels (colorimetric kit (**A**) and high-performance liquid chromatography (**B**,**C**)), mitochondrial activity (**D**; flow cytometry of mitotrackers red vs. green) and ATP production (**E**, bioluminescence kit) at 24 h and 48 h post-treatment. * *p* < 0.05, *** *p* < 0.005 and **** *p* < 0.001 for AZD3965 treatment versus control condition.

Extracellular lactate levels were assessed in culture medium collected at 24 and 48 h post-AZD3965 treatment to evaluate the effectiveness of AZD3965 in the disruption of lactate transport. The assessment of lactate production by an enzymatic kit (Figure 2A) and by HPLC (Figure 2B) revealed, in both assays, that HT1376R cells had their lactate levels decreased significantly and in a dose-dependent manner at both time points. This pattern was not observed in 253J cells; these cells showed constant extracellular lactate levels despite AZD3965 treatment. 

The assessment of extracellular glucose levels by HPLC (Figure 2C) in the AZD3965-treated HT1376R cells showed a significant increase at the highest AZD3965 concentrations, especially at 48 h of treatment. This indicates that glucose consumption was inhibited by AZD3965 treatment. The same tendency was observed in 253J cells, although the results were not significant. As expected, lower levels of glucose were obtained with both cell lines (but mainly with 253J cells) upon 48 h of AZD3965 treatment, when compared to 24 h, which is indicative of glucose consumption in a time-dependent manner. We additionally checked the expression levels of the glycolysis rate-limiting enzymes HK2 and PFKL (phosphofructokinase, liver type) at 48 h of treatment, but no significant differences among control and treatment conditions were obtained (Figure S3).

Mitochondrial activity (Figure 2D) and ATP production (Figure 2E) were also assessed in the control versus AZD3965-treated cells as additional assessments of the metabolic functions of the cells; non-significant fluctuations of both parameters were obtained in both cell lines for the two assays.

**Figure 3:**
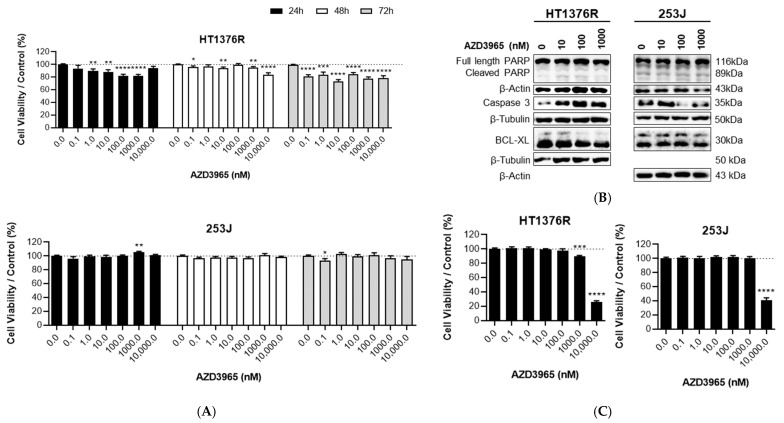
Effect of AZD3965 on the viability of HT1376R and 253J urothelial bladder carcinoma cell lines, detected by the Sulforhodamine B assay at 24, 48 and 72 h post single-dose treatment (**A**) and at 24 h post triple-dose treatment (given at 24 h, 48 h and 72 h post cells’ incubation, (**C**)), and by Western blot analysis of proteins associated with cell death pathways at 24 h post-treatment (**B**). Western blot (**B**) is representative of similar blots from three independent cell lysates (shown in Figure S4; quantification of the results shown in Figure S5); β-Actin and β-Tubulin were used as loading controls. * *p* < 0.05, ** *p* < 0.01 *** *p* < 0.005 and **** *p* < 0.001 for AZD3965 treatment versus control condition.

To evaluate the effect of AZD3965 on the viability of UBC cells, HT1376R and 253J cells were treated with increasing concentrations of the compound for 24, 48 and 72 h (Figure 3A). Generally, AZD3965 had a limited although significant effect on the viability of HT1376R cells, which was more evident at 72 h and independent of the dose. For these cells and at this time-point, an increase in the expression of cleaved PARP (poly ADP-ribose polymerase) and caspase 3, and a decrease in BCL-XL levels, was noted (especially for 100 and 1000 nM) in the Western blot results (Figure 3B; quantification of the results in Figure S4), indicating a possible induction of cell death upon treatment through the intrinsic pathway of the apoptotic cascade. Inversely, 253J cells seemed to be resistant to treatment, especially for higher dosages. No alterations in the expression prolife of full and cleaved PARP were observed for 253J cells, while a decrease in caspase 3 levels at 100 and 1000 nM was noted.

An assessment of the effect of daily doses of AZD3965 on cell viability was also performed (Figure 3C). Thus, with the same range of concentrations used before (0.1–10,000 nM of AZD3965), cells were treated daily for three consecutive days (treatment at 24, 48 and 72 h post incubation). For both cell lines, this modality of treatment did not affect cell viability except for the highest doses (1000 and 10,000 nM) in HT1376R cells and for 10,000 nM in 253J cells. The abrupt reduction in viability observed for AZD3965 10,000 nM in both cell lines suggests a strong cytotoxic effect.

**Figure 4:**
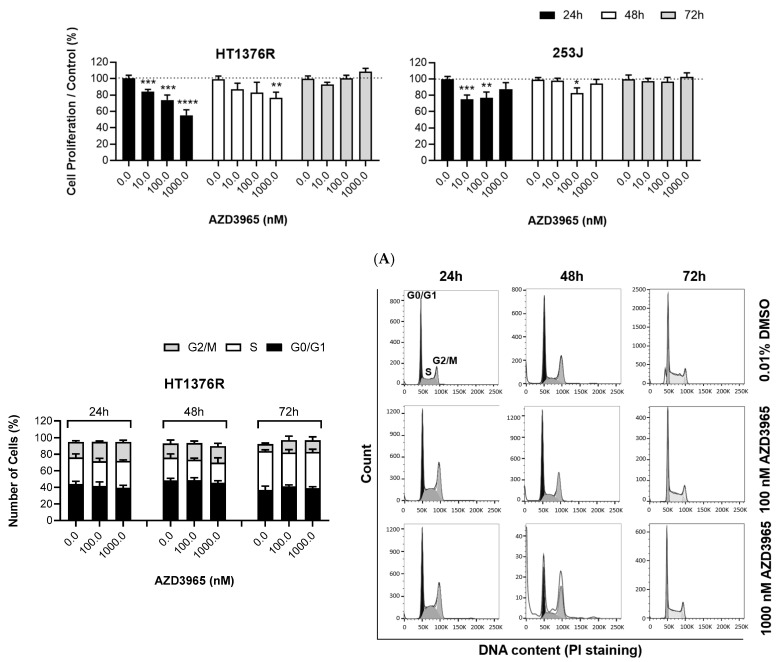

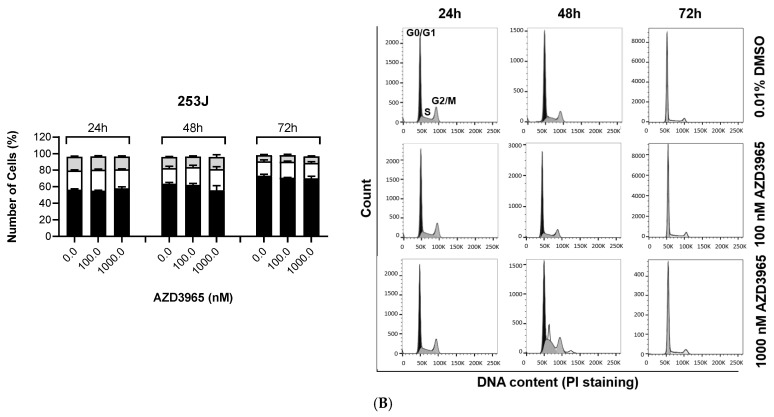
Effect of AZD3965 on the proliferation of HT1376R and 253J urothelial bladder carcinoma cell lines, detected by the BrdU incorporation assay (**A**) and by quantification of the cells in distinct phases of the cell cycle by flow cytometry analysis of propidium iodide (PI) (**B**) at 24, 48 and 72 h post-treatment. In (**B**), a representative cell cycle profile of each condition is shown. * *p* < 0.05, ** *p* < 0.01 ***, *p* < 0.005 and **** *p* < 0.001 for AZD3965 treatment versus control condition.

Cell proliferation upon AZD3965 treatment was evaluated in HT1376R and 253J cell lines with BrDU staining. The drug affected proliferation in both cell lines (Figure 4A). A significant decrease in proliferation was observed in HT1376R cells, in a dose-dependent manner, for both 24 and 48 h treatment; at 72 h, cells generally restored their normal proliferation rate. Regarding 253J cells, the effectiveness of the drug, although significant at some concentrations, was more limited than in HT1376R cells. At 72 h, the resistant phenotype observed for HT1376R cells was also observed for this cell line. 

AZD3965 impact on the cell cycle profile was evaluated with PI staining using flow cytometry (Figure 4B). HT1376R showed a tendency for the accumulation of cells at the G2/M phase, especially at 24 of treatment, possibility suggesting cell cycle arrest at these phases. These results, although not significant, are coherent with the higher halt of cell proliferation observed with the BrDU assay. Cell cycle analysis for 253J cells demonstrated that no major alterations resultant of treatment occurred, although a tendency for an increased number of cells at the S phase at 100 nM for 24 h of treatment is perceptible, as well as for 1000 nM at 48 h of exposure.

**Figure 5:**
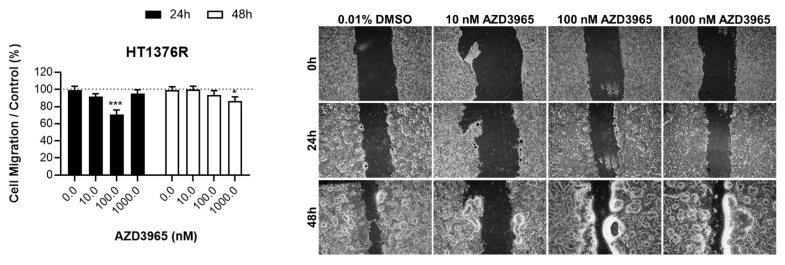

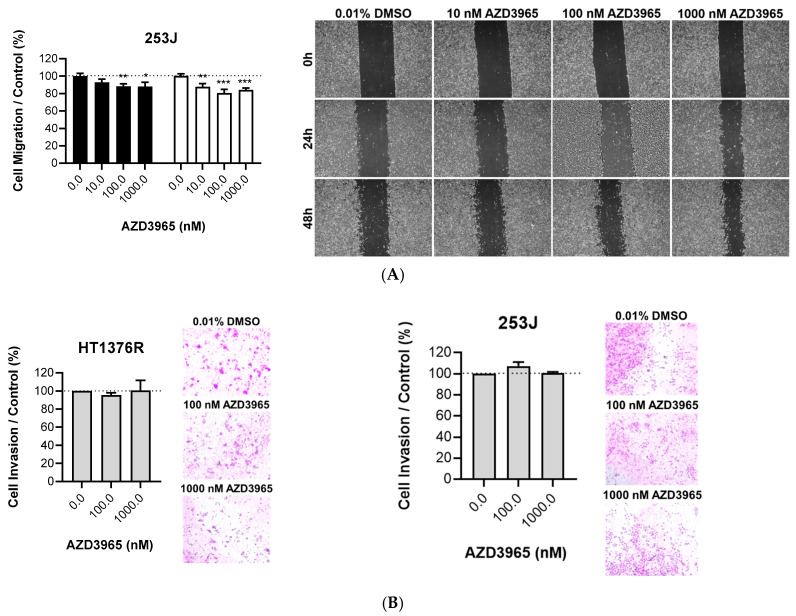
Effect of AZD3965 on the migration (**A**) and invasion (**B**) abilities of HT1376R and 253J urothelial bladder carcinoma cell lines, detected by the wound-healing assay at 24 and 48 h post-treatment (**A**), and by matrigel invasion chambers at 72 h post-treatment (**B**). Representative pictures of the assays for each condition are shown. * *p* < 0.05, ** *p* < 0.01 and *** *p* < 0.005 for AZD3965 treatment versus control condition.

The migration ability of UBC cells treated with 10, 100 and 1000 nM of AZD3965 was tested at 24 and 48 h of treatment (Figure 5A). HT1376R cells showed a limited response to AZD3965, while the migration of 253J cells was significantly affected by the drug, in a time- and dose-dependent manner. AZD3965 higher doses were selected to determine its effect on invasion ability (Figure 5B); in this assay, no significant differences were noted between the control and treatment conditions, for both cell lines.

### 3.2. Impact of MCT1 Inhibition by AZD3965 in an “In Vivo” Muscle-Invasive UBC Model

**Figure 6:**
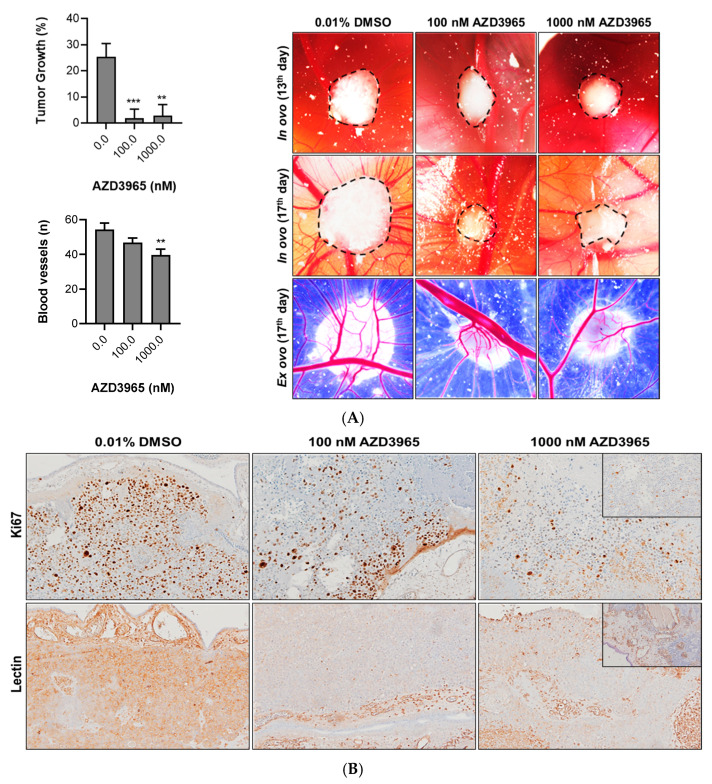
Effect of AZD3965 on tumor growth and angiogenesis of HT1376R urothelial bladder carcinoma cell lines “in vivo”, detected by the chicken chorioallantoic membrane (CAM) assay at 96 h post-treatment. In (**A**), quantification of tumor growth and blood vessels formation is shown, followed by representative images at 13th (“in ovo”—start of treatment) and 17th (“in ovo” and “ex ovo”—end of treatment) days post egg incubation (4th and 8th days post cells’ injection, respectively) of the control (0.0 nM AZD3965, 0.01% DMSO) and treated (100.0 and 1000.0 nM AZD3965) conditions. In (**B**), representative images of the excised CAM tissue sections of each condition, immunostained for lectin and Ki67, are shown. Positive controls in insets (CAM section with known positivity for lectin; lymphoma section with known positivity for Ki67). Original magnifications of 100 or 200×. ** *p* < 0.01 and *** *p* < 0.0005 for AZD3965 treatment versus control condition.

The evaluation of AZD3965 activity “in vivo” was performed using the CAM model assay (Figure 6A). Taking into consideration the results obtained in the “in vitro” functional assays, the HT1376R cell line was chosen for the “in vivo” studies. Cells were grafted into the CAM to allow for tumor formation and development. Tumors were then treated with 100 nM (*n* = 15) and 1000 nM (*n* = 16) of the compound. AZD3965 significantly reduced tumor growth at both doses. Generally, tumors had their growth rate slowed down, although some had their size diminished. Additionally, blood vessel formation was assessed, and treatment promoted a decrease in the angiogenic ability of tumors in a dose-dependent manner. Excised tumors were then fixed, and tissue sections were obtained for the immunohistochemistry evaluation of proliferation and angiogenesis markers (Figure 6B). Staining with Ki67 showed a reduction in proliferative cells in the treated tumors, and lectin staining confirmed the reduction in blood vessel formation induced by AZD3965 “in vivo”.

### 3.3. Impact of AZD3965 and Cisplatin Combined Therapy in Muscle-Invasive UBC Cells

**Figure 7:**
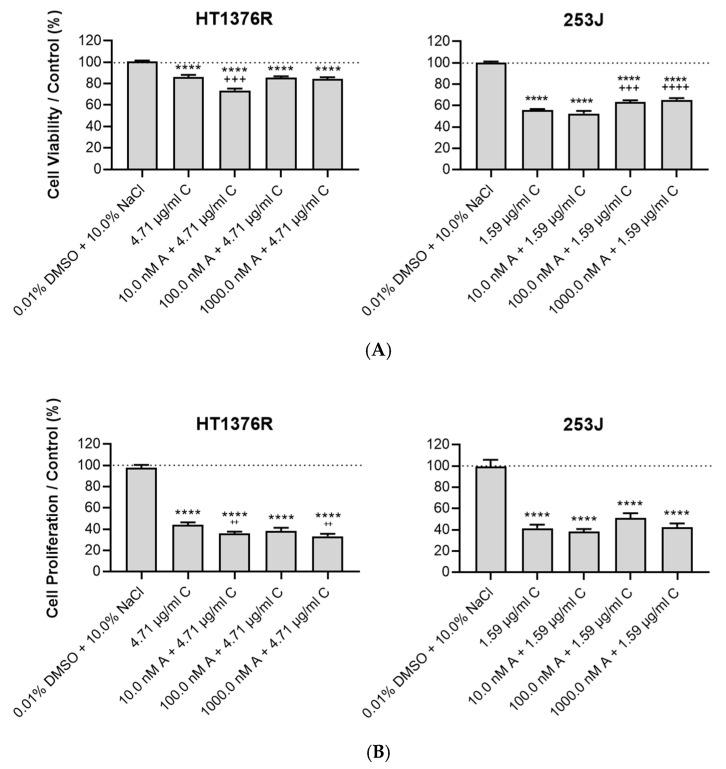
Effect of cisplatin (C), administered alone and in combination (at indicated dosages) with AZD3965 (A), on the viability (**A**) and proliferation (**B**) of HT1376R and 253J urothelial bladder carcinoma cell lines, detected by the Sulforhodamine B assay (**A**) and by the BrdU incorporation assay (**B**) at 72 h post-treatment. **** *p* < 0.0001 for cisplatin treatment (alone or combined) versus control condition. ^++^ *p* < 0.01, ^+++^ *p* < 0.005 and ^++++^ *p* > 0.001 for combined treatment (at indicated dosages) versus cisplatin treatment.

To evaluate the impact of AZD3965 treatment on cisplatin response in HT1376R and 253J cell lines, cell viability was assessed for selected concentrations of AZD3965 (10, 100 and 1000 nM) administered in combination with cisplatin, during 72 h of treatment (Figure 7A). The results showed that for the HT1376R cell line, viability was significantly reduced, in comparison to the vehicle alone, when 4.71 µg/mL cisplatin was administered to the cells (20% of viability reduction). AZD3965 sensitized the cells to cisplatin effects, especially with the combination 10 nM AZD3965 + 4.71 µg/mL cisplatin, in which a significant impact on cell viability was noted when compared to cisplatin alone. Regarding 253J cells, cisplatin reduced viability by about 50% when compared to the control condition. Combining 10 nM AZD3965 with 1.59 µg/mL cisplatin did not have any significant effect on the cells when compared to cisplatin alone, and at higher concentrations AZD3965 induced a general improvement in cell viability upon combination with cisplatin. This suggests that AZD3965 antagonizes cisplatin effects in these cells.

We also evaluated cell proliferation upon the previously referred conditions (Figure 7B). Cisplatin significantly reduced the proliferative abilities of both HT1376R and 253J cells (by about 50–60%). Importantly, combining 4.71 µg/mL cisplatin with 10 or 1000 nM AZD3965 promoted a significant reduction in proliferation when compared to cisplatin alone in HT1376R cells. In opposition, no differences where obtained in 253J cells when AZD3965 was combined with 1.59 µg/mL cisplatin, with AZD3965 inducing slight increases in proliferation upon different combinations, which corroborates the antagonic role of AZD3965 in cisplatin effectiveness.

## 4. Discussion

In this study, we aimed to characterize the activity of AZD3965 in urothelial bladder cancer cells. AZD3965 specifically targets MCT1, with lower affinity for MCT2 and no affinity for MCT4. As mentioned above, cancer cells rely on MCTs to extrude lactate and avoid intracellular acidosis, which makes them attractive targets for cancer therapy. 

Pharmacological inhibition of MCT1 with AZD3965 has been tested in different malignancies. Despite its high affinity for MCT1, and the ability to impair cell viability and proliferation in multiple cell lines [26,27,33,34,35], the effect of this compound is not transversal. In fact, treatment failed in some studies, and researchers have suggested that the lack of response is due to compensation of MCT1 activity [27,36,37]. It was found that cell lines overexpressing MCT4 would present a resistant phenotype to MCT1 inhibition with AZD3965 as cells seemed to engage in a compensatory mechanism to promote lactate exportation and avoid cell death. In our study, and having these findings in mind, we chose to test the activity of AZD3965 in two cell lines with different MCT4 expression patterns: HT1376R, with low MCT4 levels, and 253J, with an inverse phenotype. The concentration range chosen to test AZD3965 effects on extracellular lactate levels and cell viability (0.1–10,000 nM) was shown to be effective when tested in breast [29] and lymphoma malignant cells [38]. Following the results obtained in the previously referred to experiments, drug concentrations that were seen to have a more pronounced effect were chosen for further testing. 

HT1376R cells released less lactate to the extracellular medium upon AZD3965 treatment when compared to the control condition in both colorimetric and HPLC assays (dose dependent), confirming what has been previously observed in breast cancer cells [34]. For 253J cells, extracellular lactate levels were practically unaltered by AZD3965 treatment when compared to the control group, which leads us to hypothesize that the induction of resistance to AZD3965 treatment promoted by MCT4 overexpression might also occur in the UBC setting. On the other hand, we found that HT1376R cells had their glucose consumption rates decreased for the highest compound doses, and the same tendency (although not significant) was observed for 253J cells. In a previous study, Beloueche-Babari reported similar results when analyzing ^13^C-glucose levels by NMR spectroscopy [39]. Despite these changes in lactate extrusion and glucose uptake, no alterations in the expression levels of HK2 and PFKL upon treatment were observed, which could indicate that AZD3965 does not strongly affect the glycolytic cascade in MI-UBC cells. In recent years, some studies reported that treatment with this compound could promote a shift in the metabolic process in cancer cells. In fact, the study by Beloueche-Babari et al. [39] showed that AZD3965 treatment enhanced the TCA cycle, which contributed to the maintenance of cell viability. However, another study reported that AZD3965 disrupted both the glycolytic and the TCA cycle routes through PDH inhibition, which considerably delayed tumor growth in an “in vivo” model that utilized lactate for TCA metabolism [34]. Herein, we found no alteration regarding mitochondrial activity and ATP production in both cell lines, possibly indicating that HT1376R and 253J cells could indeed be maintaining glycolysis as a standard energy production source. 

AZD3965 did not seem to affect the viability of these cells. On the other hand, HT1376R cells’ viability was significantly affected by AZD3965, especially after 72 h of treatment, but only a maximum of 30% reduction was obtained. For this time-point, we observed a tendency for increased cleaved PARP with treatment, as well as an increase in the expression of caspase 3. Cleavage of PARP induced by caspase 3 was shown to be an indicator of late apoptotic stages [40], and our results seem to point that cell fate upon exposure to AZD3965 might be related to the intrinsic apoptotic pathway. Furthermore, a small decrease in the expression of BCL-XL, an anti-apoptotic member of the Bcl-2 family that regulates cell death and is generally upregulated in cancer, is perceptible. A decrease in the expression of caspase 3 in AZD3965-treated 253J cells corroborates that these cells overcome the treatment effect, sustaining cell viability, which is also confirmed by the lack of changes in the expression profile of PARP and BCL-XL. Similar effects regarding apoptosis signaling under low MCT4 expression have already been observed [33,41], although other studies report that cell death with this compound may be undertaken through different routes (as autophagy or even necrosis) [27,39]. Once a single dose of AZD3965 did not result in a pronounced effect on cell viability (reductions of about 20–30% when comparing to control condition), we attempted a different modality, according to the study by Benyahia et al. [29], in which cells were exposed to daily doses of the compound for 72 h. This approach had a strong cytotoxic effect, especially in HT1376R cells, when higher doses of the compound were used (1000 and 10,000 nM).

It was demonstrated that MCT1 inhibition promotes a reduction in the proliferative ability of cancer cells [42,43]. In our study, AZD3965 impaired the proliferation of HT1376R cells at 24 and 48 h of treatment, especially for higher doses. Interestingly, the compound was also able to decrease the proliferation for 253J cells but for lower doses, suggesting that higher doses may reduce sensitivity to the anti-proliferative potential of the drug in these cells. No anti-proliferative effect was observed in both cell lines when treatment was extended to 72 h. Regarding the implications on cell cycle regulation, AZD3965 seems to tendentially promote cell cycle arrest in the G2/M phase in the HT1376R cell line, especially at 24 h of treatment, with this effect being reduced upon longer exposure times, suggesting that cells tend to adapt its cell cycle regulation to the presence of the inhibitor in order to avoid severe damage. These results, although not significant, seem to be in line with the inhibition of the proliferative rate seen with the BrDU assay, although a more pronounced effect on cell cycle would be expected for this lowest timepoint. In other studies, the knockdown of MCT1 and its pharmacological inhibition with AR-C155858 induced cell cycle arrest at G0/G1 phase [44,45].

The migration and invasion of cancer cells are intrinsically associated to the development of metastasis [46]. The overexpression of MCT1 and MCT4 seems to actively contribute to these aggressiveness features, and the disruption of MCT1 activity leads to the impairment of both properties [23,47,48]. Sheng et al. reported that treatment with AZD3965 in osteosarcoma cell lines markedly reduced the expression of metastasis-associated proteins [49]. Interestingly, a recent report indicated that the opposite occurs in breast tumor models, where treatment with AZD3965 has led to lung metastasis formation [34]. In our study, both AZD3965-treated cells had their migration abilities decreased, but no effect was observed in either cell line regarding invasion capacity, suggesting that the influence of AZD3965 treatment in bladder cancer metastasis should be limited. Possibly, the disruption of MCT1 alone is insufficient to compromise metastatic potential in this model. The close association of CD147 (chaperone of both MCT1 and MCT4) with metastasis [50,51,52], as well as the involvement of multiple other proteins of the metastatic cascade [46], are probably playing a major role here.

As mentioned, AZD3965 is currently in a Phase I/II clinical trial for advanced solid tumors and lymphomas, and is orally available [24,25]. Many studies have evaluated its pharmacokinetics and effectiveness in “in vivo” models [28]. Some authors have reported that the drug is able to reduce tumor growth, tumor volume and intratumoral lactate levels [26,27,29,36,37,44,53]. Herein, we used the CAM model assay to evaluate the efficacy of AZD3965 in an “in vivo” bladder cancer model. HT1376R tumors were treated with 100 and 1000 nM of the compound. AZD3965 retarded tumor growth, as a significant decrease in tumor size was observed. Despite these results, we have not observed tumor regression. A dose-dependent reduction in blood vessels formation was also observed, and the immunohistochemistry of tissue samples confirmed a decrease both in the proliferative status (as observed “in vitro”) and the angiogenic capacity of tumor cells. 

Finally, we intended to test if the combination of AZD3965 with cisplatin, the gold-standard chemotherapeutic agent for MI-UBC patients, would improve the response to this classical treatment. Patients with advanced metastatic disease or unsuitable for surgery usually undergo treatment with cisplatin-based agents but are highly susceptible to chemoresistance [16]. Combination studies using AZD3965 demonstrated that this drug is able to enhance radiosensitivity [26] but also significantly impairs tumor growth in combination with other agents when compared to the effect of either drug alone [36,38,54]. Thus, we combined AZD33965 with cisplatin and evaluated their efficacy in impairing cell viability and proliferation. Combination schemes did not generally overperform the activity of cisplatin alone, with the exception of 10 nM of AZD3965 that, combined with cisplatin, was able to sensitize HT1376R cells to cisplatin and significantly decrease their viability when compared to chemotherapy alone. Proliferation was also impaired at 10 and 1000 nM AZD3965 with cisplatin. Thus, it seems that AZD3965 may be able to sensitize MCT4-low-expressing cisplatin-resistant UBC cells to the cytotoxic and cytostatic effects of the drug. It would be interesting to perform drug combination studies with lower doses of cisplatin. If the same sensitizing effect was achieved, that would imply not only an improved therapeutic profile but also a reduction in cisplatin doses, likely ameliorating its severe, dose-limiting adverse effects [55]. Apart from that, the opposite effect was observed in 253J cells. AZD3965 seemed to antagonize cisplatin effects regarding both the viability and proliferative abilities of the MCT4 high-expressing 253J cell line. Thus, these studies seem to indicate that the combination of AZD3965 with cisplatin does not offer a solution to potentiate cisplatin effects in MCT4 high-expressing bladder cancer.

In summary, the results of the present study seem to indicate that UBC cells are generally not sensitive to AZD3965, especially under MCT4 overexpression. Although MCT1 presents higher affinity for lactate than MCT4 [56], the loss or reduced functionality of MCT1 may lead cancer cells to use MCT4 for lactic acid extrusion to prevent cell death. Inhibiting both isoforms at the same time could possibly have a major impact on treatment response, but this scenario must be looked at with caution once MCT1 and MCT4 are widely expressed in the human body, which could make their simultaneous blockage problematic to the normal function of many tissues [57]. Although this seems to be a plausible explanation to the lack of response to AZD3965, other proteins may be involved in the resistant phenotype. Despite being a specific MCT1 inhibitor, AZD3965 has a six-fold lower affinity for MCT2, which possibly means that cells that overexpress this isoform—which seems to be the case of 253J cells—could also be more resistant to AZD3965 [58,59]. Moreover, although MCTs play a crucial role in the regulation of lactate shuttling to avoid its intracellular accumulation and a decrease in intracellular pH, other proteins are also responsible for maintaining the pH balance within a cell. These pH regulators, such as CAIX, may also play a role in the cells’ sensitivity to AZD3965. CAIX is overexpressed in UBC and has been associated with high recurrence rates [60,61]; it seems to facilitate monocarboxylate transportation by binding to CD147, thus enhancing MCT1/4 activity [62]. Thus, although AZD3965 may decrease lactate exportation through MCT1 targeting, this may not result in a significant decrease in intracellular pH due to CAIX interference in the pH balance.

This study provides the first preclinical evaluation of the effect of AZD3965 in muscle-invasive bladder cancer. We used cell lines with distinct phenotypes regarding MCT4, which allowed us to conclude that MCT4 is potentially one the most important players in AZD3965 resistance in UBC. Thus, further studies should be performed to validate the anticancer effects of AZD3965 in bladder tumors expressing low levels of MCT4, namely, advanced functional assays and “in vivo” experiments with mouse xenografts, but also to dissect the resistance mechanism underlying treatment with this compound in UBC. For instance, a response to dual genetic/pharmacological MCT1/MCT4 inhibition would be important to validate our hypothesis. It would also be important to further explore the mechanistic aspects of AZD3965 impact in bladder cancer metabolism, for which gold standard assays analyzing glycolysis and mitochondrial activity (e.g., seahorse analysis, ^1^H NMR-based metabolomics) would be useful. Moreover, it is well known that metabolic heterogeneity and lactate shuttles between cancer cells and among cancer and non-malignant associated stromal cells occur within the tumor microenvironment [63], including in bladder cancer [15]. Thus, the response to AZD3965-induced MCT1 inhibition in co-culture systems (UBC cells with cancer associated fibroblasts or endothelial cells) is suggested as a starting point to study AZD3965 effects under a broader and realistic scenario.

## 5. Conclusions

The present study demonstrates that AZD3965 has a limited effect on bladder cancer treatment. Its effectiveness seems to be strongly associated with the low expression of MCT4, being able to impact several functional cell properties in this setting as well as delay tumor growth “in vivo”. In combination with cisplatin, AZD3965 was able to sensitize cisplatin-resistant UBC cells to the chemotherapeutic agent. On the other hand, cells expressing high levels of MCT4 showed general resistance to AZD3965 treatment, and no benefit was obtained when combining this drug with cisplatin. To the best of our knowledge, this was the first study to assess the activity of AZD3965 in muscle-invasive UBC. However, further studies are needed to validate its usefulness in UBC models where MCT4 levels are low and to dissect the resistance mechanism in models that show high MCT4 expression.

## Data Availability

All of the data generated during the study are included in the article. Further enquiries can be directed to the corresponding author.

## Supplementary Materials

The following supporting information can be downloaded at https://www.mdpi.com/article/10.3390/pharmaceutics15122688/s1, Table S1. The list of antibodies used in Western Blot, immunofluorescence and immunohistochemistry; Figure S1. The replicate blots for the quantification of the Western blot shown in Figure 1A; Figure S2. The quantification results of the replicate blots shown in Figure S1; Figure S3. The replicate blots for quantification of the Western blot shown in Figure 3B; Figure S4. The quantification results of the replicate blots shown in Figure S3. * *p* < 0.05 and ** *p* < 0.01 for AZD3965 treatment versus the control condition.
