# Supplementary material for: Effects of Lactate Transport Inhibition by AZD3965 in Muscle-Invasive Urothelial Bladder Cancer
Source: Pharmaceutics. 2023 Nov 28;15(12):2688. doi: 10.3390/pharmaceutics15122688 (PMC10747642; doi:10.3390/pharmaceutics15122688)
Supplement: Supplementary file 1 [file pharmaceutics-15-02688-s001.zip › pharmaceutics-2493807-supplementary.pdf]

Table S1. List of antibodies used in Western Blot, immunofluorescence and immunohistochemistry

	Antibody	Company	Reference	Dilution
		**Western Blot**		
**Primary antibodies**	MCT1	Santa Cruz Biotechnology®	sc-365501	1:500
	MCT4	Santa Cruz Biotechnology®	sc-50329	1:500
	CD147	Santa Cruz Biotechnology®	sc-46700	1:500
	LDHA	Santa Cruz Biotechnology®	sc-100775	1:1000
	HK2	AbCam	ab104836	1:2000
	PFKL	AbCam	ab37583	1:100
	CAIX	AbCam	ab15086	1:2000
	Caspase 9	Cell Signaling® Technology	#9502	1:1000
	p53	Cell Signaling® Technology	#2527	1:1000
	Bcl-xL	Santa Cruz Biotechnology®	sc-8392	1:300
	PARP	Cell Signaling® Technology	#9542	1:500
**Secondary antibodies**	m-IgGκ BP-HRP	Santa Cruz Biotechnology®	sc-516102	1:1000
	IgG-HRP	Santa Cruz Biotechnology®	sc-2357	1:1000
**Loading controls**	β-actin	Santa Cruz Biotechnology®	sc-8432	1:500
	β-Tubulin	AbCam	ab15246	1:2500
	GAPDH	Santa Cruz Biotechnology®	1:1000	1:1000
		Immunofluorescence		
**Primary antibodies**	MCT1	AbCam	ab35944	1:100
	MCT4	Chemicon®, Sigma-Aldrich®	AB3316P	1:100
	CD147	Santa Cruz Biotechnology®	sc71038	1:500
**Secondary antibodies**	Alexa Fluor® 488	Invitrogen™	A11008	1:500
	Alexa Fluor® 594	Invitrogen™	A11032	1:500
		Immunohistochemistry		
**Primary antibodies**	Ki-67	AbCam	ab16667	1:250
	Lectin	Vector Laboratories	B1305	1:100

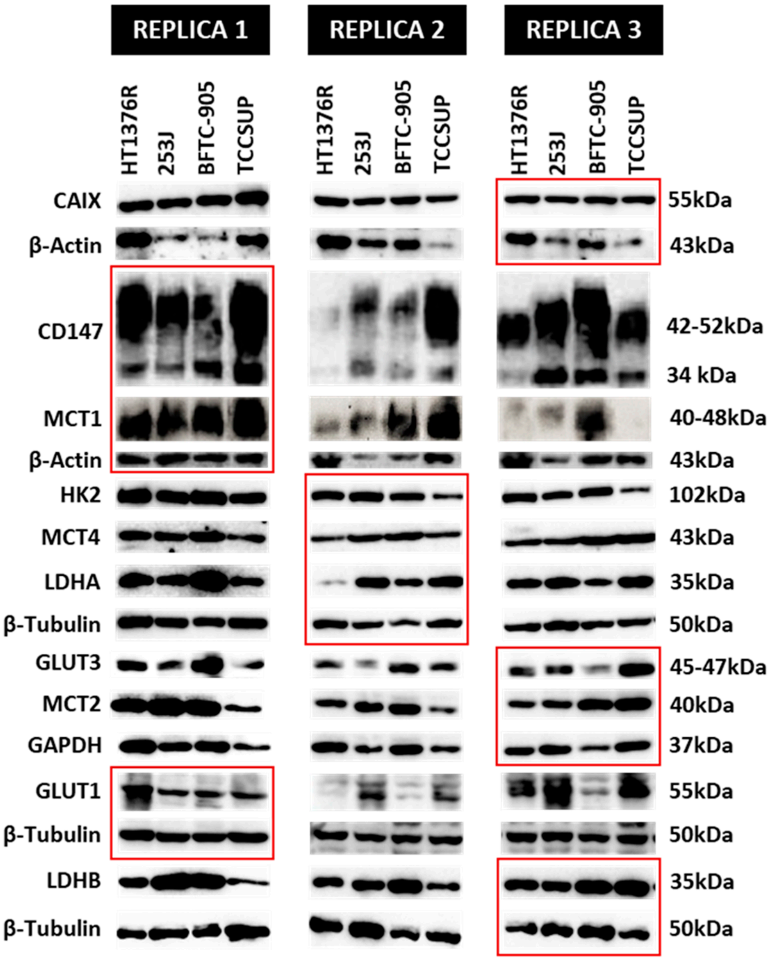

Figure S1. Replicate blots for quantification of the Western blot shown in Figure 1A.

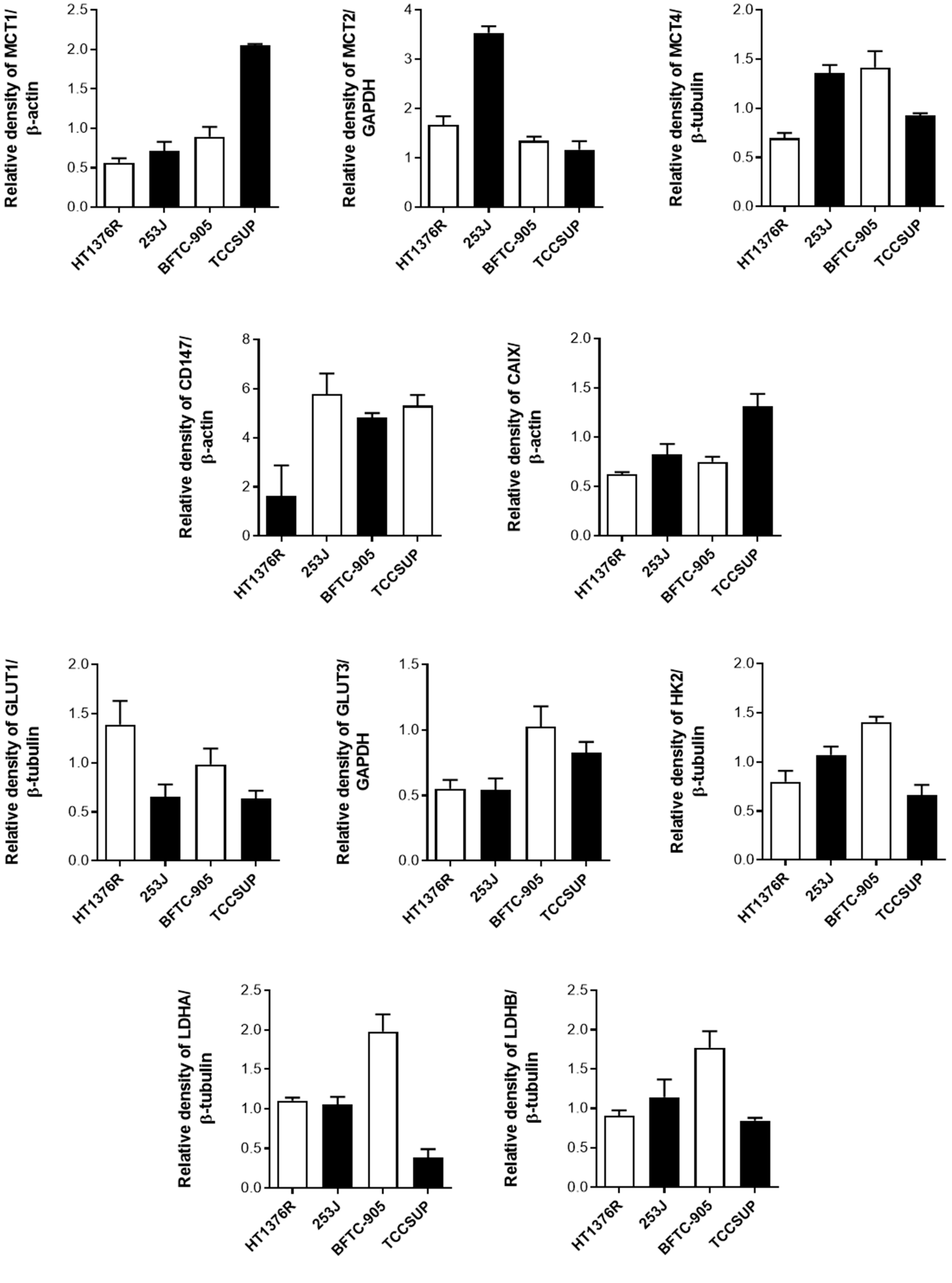

Figure S2. Quantification results of the replicate blots shown in Figure S1.

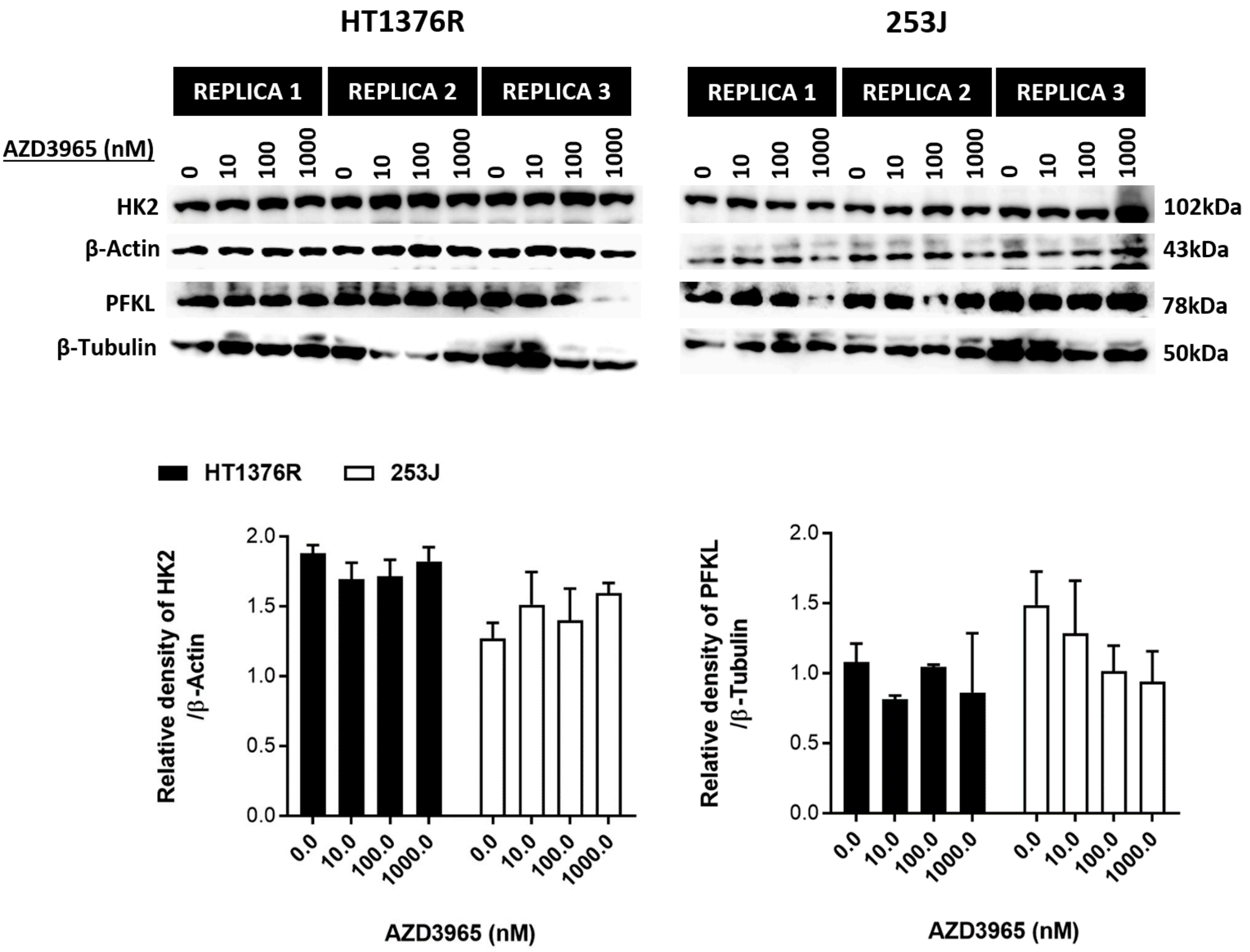

Figure S3. Replicate blots (from three independent cell lysates) and quantification results of the Western blot showing levels of HK2 and PFKL in HT1376R and 253J urothelial bladder carcinoma cell lines upon treatment with AZD3965 for 48h; β-Actin and β-Tubulin were used as loading controls.

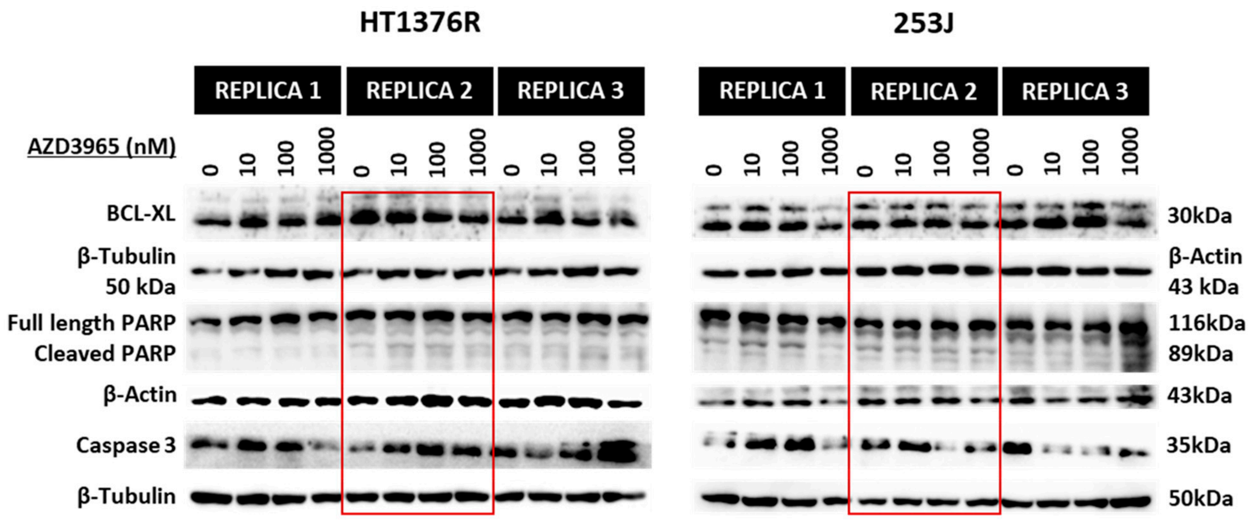

Figure S4. Replicate blots for quantification of the Western blot shown in Figure 3B.

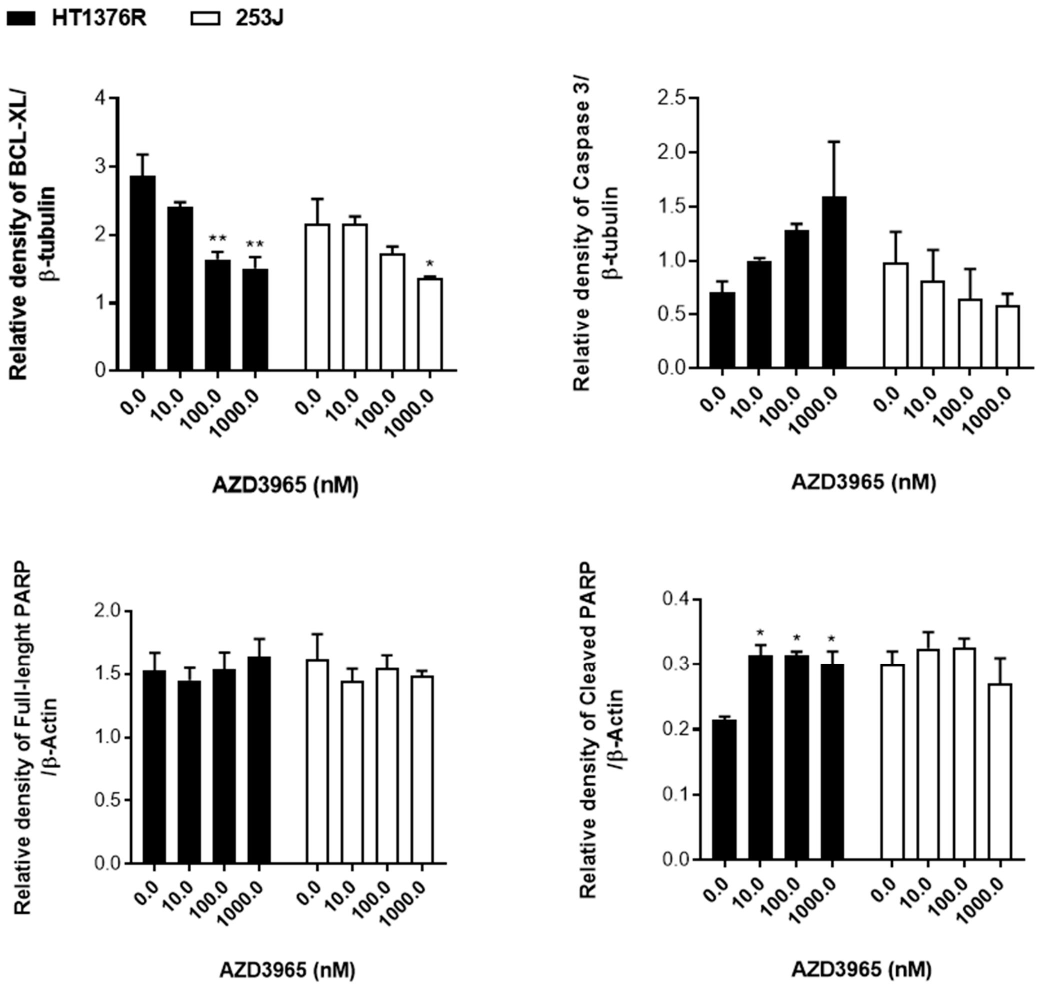

Figure S5. Quantification results of the replicate blots shown in Figure S4. *p<0.05 and **p<0.01 for AZD3965 treatment *versus* control condition.
